# The Pathology of Type II Modic Changes: Fat Deposition or Osteosclerosis? A Study Using CT Scan

**DOI:** 10.1155/2018/6853720

**Published:** 2018-06-04

**Authors:** Chi Sun, Hongli Wang, Jianyuan Jiang, Feizhou Lu, Xiaosheng Ma, Xinlei Xia

**Affiliations:** ^1^Department of Orthopedics, Huashan Hospital, Fudan University, Shanghai, China; ^2^The Fifth People's Hospital of Shanghai, Fudan University, Shanghai, China

## Abstract

**Study Design:**

A retrospective cross-sectional study of type II Modic changes (MCs).

**Objective:**

To evaluate the CT values of type II MCs and determine their relationship with disc degeneration.

**Methods:**

124 type II MCs from 66 patients' MRI and CT were included and analyzed. Disc degeneration adjacent to MCs was evaluated based on Pfirrmann classification. CT values of bone marrow area and endplate from MC regions, adjacent non-MC regions, and L1 vertebra were measured. CT value changes (ΔCT value) were defined as MCs' CT value minus non-MCs'. According to the types of variables, paired t-test, signed-rank test, two-way ANOVA, and Friedman test were used.

**Results:**

The CT value of MCs was significantly higher than that of non-MCs at both bone marrow area and endplate (P<0.0001, resp.). Good consistency was found between non-MCs and L1 vertebra in the CT value of bone marrow area and endplate (P=0.2129, P=0.2272, resp.), suggesting the control group was valid. Adjacent to type II MCs, there were 8 (6.4%) discs with grade III degeneration, 58 (46.8%) with grade IV, and 58 (46.8%) with grade V. The ΔCT value of grade V disc degeneration was larger than that of grade IV at the endplate (P=0.0150).

**Conclusions:**

Osteosclerosis may exist in type II MCs. The more severe the degeneration of the adjacent disc, the greater the degree of osteosclerosis.

## 1. Introduction

Modic changes (MCs) are common on lumbar spine MRI and even sometimes on the cervical or thoracic spine. MCs are lesions of the bone marrow area and endplate and are thought to be associated with intervertebral disc degeneration (IDD). The prevalence rates of MCs range from 0.5% to 62% [1–3]. First found by de Roos et al. [4], MCs were classified by Modic et al. into three types [5, 6]. Traditionally, type II MCs (high T1W and T2W signals) are regarded as a stable and chronic fat deposition. While type I MCs (low T1W and high T2W signals) are thought to indicate inflammatory reactions, type III (low T1W and T2W signals) indicates osteosclerosis.

MCs have been studied for years. While type II MCs are thought to be relatively stable lesions, their relationship with clinical symptoms is unclear. Some reports believe that type II MCs could cause low back pain while other reports showed no correlation [7–10]. As for the kinematical characteristics of MCs, Fan et al. thought type II MCs might increase angular or translational motion in segments, which was previously believed to be stable [11]. Although the real pathological process of these MCs remains unclear, Feng et al. found not only fat deposition but also inflammation in type II MCs by using an MR fat suppression sequence [10]. Kuisma et al. reported that some sclerosis could be visualized on CT scans in the area of type II MCs [7]. However, their study focused on the lower lumbar spine and evaluated the CT features of the bone marrow area of MCs, ignoring the endplate. Additionally, the relationship between sclerosis and disc degeneration was not analyzed.

To date, most studies on MCs have been based on MRI by T1-weighted image (T1WI), T2-weighted image (T2WI), and fat suppression sequence, and few were conducted with CT scans. While MRI can show kinds of different tissues via various T1W and T2W signals, CT provides vivid bony structure image and precise CT value of tissues. We hypothesized that a specific kind of pathological process, not only fat deposition, in type II MCs would show a particular CT value. Thus, our study aimed to evaluate the CT values of type II MC regions, find their association with disc degeneration, and speculate about the real pathological process.

## 2. Materials and Methods

### 2.1. Study Population

The population of this study consisted of patients coming to our hospital to receive lumbar spine surgery from December 2013 to June 2015. All routine preoperative imaging data (MRI and CT) were retrospectively reviewed by one author (H.W.) for the presence of any type of MC. Patients were included if there were MCs in the lumbar spine on MRI. Patients with type I, type III, or mixed MCs (I and II or II and III) were excluded. Meanwhile, suspected metastatic lesions, scoliosis, lumbar spine infections, fresh vertebral fractures, ankylosing spondylitis, lumbar instrumentation, any lesions in L1 vertebra, and small signal changes within a corner of vertebrae were also excluded. That is, only lumbar spine MRI with visible and classic type II MCs and a healthy L1 vertebra were included for further assessment. The criteria for classic type II MCs were high T1W and T2W signals in the bone marrow area and endplate, based on the description from Modic et al. [5, 6]. Finally, a total of 66 patients were eligible. As we retrospectively assessed patients' imaging data, the study was conducted with the human subjects' understanding and consent. The Ethical Committee of Huashan Hospital affiliated to Fudan University has approved the experiments.

### 2.2. MR Imaging

MRI was performed at our hospital using a 3 T unit (Magnetom Verio, Siemens, Germany) and a phased-array torso coil. The fast-recovery fast spin-echo (frFSE) sequence was used for scanning to acquire T2W. Sagittal T2W parameters were TR/TE=1960 ms/106.3 ms, ST=4.0 mm, and matrix=512*∗*512. Sagittal T1W was obtained by a fast spin-echo (FSE) sequence, with parameters TR/TE=340 ms/12.9 ms, ST=4.0 mm, and matrix=512*∗*512.

### 2.3. CT Imaging

The CT imaging was performed using a 64-slice CT scanner (Light Speed VCT, Siemens, Germany) with a detector configuration of 64 × 1.25 mm. A standard lumbar spinal protocol with a tube voltage of 130 kV, tube current of 80-520 mA, and rotation time of 0.8 s was used. The slice thickness and reconstruction interval were 1.25 mm and 0.625 mm, respectively.

### 2.4. Qualitative and Quantitative Image Analysis

**Figure 1:**
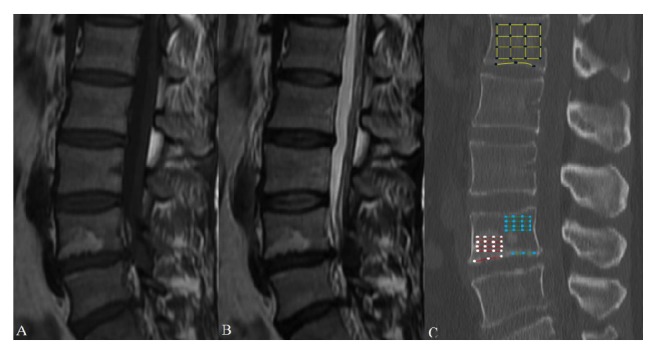
Quantitative measurements for the CT values of bone marrow area. According to the sagittal MRI plane (A for T1W, B for T2W), the same sagittal reconstructed CT scans (C) were localized on the PACS. A rectangular region of interest (ROI) as large as possible was drawn within the MC regions (red), adjacent non-MC regions (green), and L1 vertebra (yellow), which represented each bone marrow area. Points of trisection on each side were marked and connected to the opposite. The mean CT value of each group of intersection points was calculated (MCs, white; non-MCs, blue; L1 vertebra, black). A line was drawn along the endplate, and the points of trisection were marked. The mean CT value of each group of points was obtained (MCs, white; non-MCs, blue; L1 vertebra, black).

Disc degenerations adjacent to MCs from L1 to S1 were evaluated by one author (C.S.) from MR imaging data. The degree of degeneration was graded on consecutive T2W images based on the grading system of Pfirrmann et al. [12], with grades I-V referring to different degrees. CT values of the bone marrow area and endplate from MC regions, adjacent non-MC regions, and the L1 vertebra from the sagittal reconstructed CT planes were calculated. Non-MCs were defined as the healthy regions at the same vertebra where MCs presented. If both the upper and lower endplate of a vertebra had MCs, then the adjacent non-MCs would correspondingly be defined as the area with larger CT values, from the lower endplate of the superior vertebra or the upper endplate of the inferior vertebra. On the Picture Archiving and Communication System (PACS), the choice of sagittal CT plane was based on the sagittal MRI plane presenting clear MCs. If we found fine MCs on the sagittal MR plane, the location of this plane on the axial MR plane was marked. Thus, we used the same location on the axial CT plane to identify the sagittal CT plane that contained this MC. A rectangular region of interest (ROI) as large as possible was drawn on sagittal CT planes within the MC regions, adjacent non-MC regions, and L1 vertebra, which represented bone marrow area. Points of trisection on each side were marked and connected to the opposite. The mean CT value of the intersection points from the ROI was calculated and defined as the bone marrow area's CT value. For the endplate, the mean CT value of the points of trisection in the line drawn along the endplate was also obtained (Figure 1). All the CT values were measured twice by C.S. and H.W., and from these, we calculated the average, which was used in analysis.

### 2.5. Statistical Analysis

Statistical analysis was performed using STATA (version 14.0, Stata Corp LP, TX, USA). Descriptive statistics were used to describe the characteristic of study population, the number and distribution of type II MCs, and disc degeneration. Continuous variables are presented as the mean ± standard deviation (SD). CT values of bone marrow area and endplate from MC regions, adjacent non-MC regions, and the L1 vertebra were evaluated. The differences of CT values between MC regions, adjacent non-MC regions, and L1 vertebra CT values were determined using the paired t-test and Wilcoxon signed-rank test. CT values of MC regions and CT value changes (ΔCT value), defined as a MC's CT value minus the corresponding non-MC's CT value, adjacent to discs with different degrees of degeneration were compared using two-way ANOVA and the Friedman test. A p value<0.05 was considered statistical difference.

## 3. Results

### 3.1. The Number and Distribution of Type II MCs

**Figure 2:**
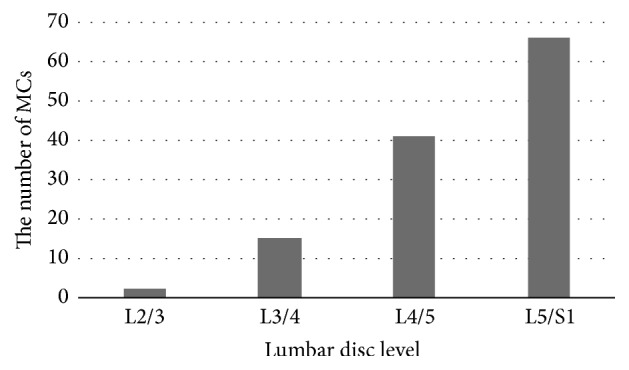
The distribution of type II MCs. As few occurred in L2/3 and L3/4, MCs were more likely to present in the lower lumbar spine, especially in L5/S1.

Our 66 patients (36 males and 30 females) had a total of 124 type II MCs. The mean age was 59.94 (41-81) years and the mean BMI was 25.05 (19.05-33.06) kg/m^2^ (Table 1). Of all the changes, 2 (1.6%) were adjacent to the L2/3 intervertebral disc, 15 (12.1%) to L3/4, 41 (33.1%) to L4/5, and 66 (53.2%) to L5/S1 (Figure 2). MCs usually presented in pairs at the same lumbar level, as shown in Table 2.

### 3.2. CT Values of MC Regions, Adjacent Non-MC Regions, and L1 Vertebra

CT values of MC regions, adjacent non-MC regions, and the L1 vertebra are displayed in Table 3. At bone marrow areas, the CT value of MCs was significantly higher than that of non-MCs (261.80±122.93 HU versus 157.28±56.21 HU, P<0.0001) and that of the L1 vertebra (261.80±122.93 HU versus 148.61±42.51 HU, P<0.0001). For the endplates, MCs also had a higher CT value than non-MCs (475.65±126.45 HU versus 402.96±109.89 HU, P<0.0001) and the L1 vertebra (475.65±126.45 HU versus 391.33±72.43 HU, P<0.0001). There was no significant difference between non-MCs and the L1 vertebra in the CT value of the bone marrow area (157.28±56.21 HU versus 148.61±42.51 HU, P=0.2129) or the endplate (402.96±109.89 HU versus 391.33±72.43 HU, P=0.2272).

### 3.3. Disc Degeneration and MCs

**Figure 3:**
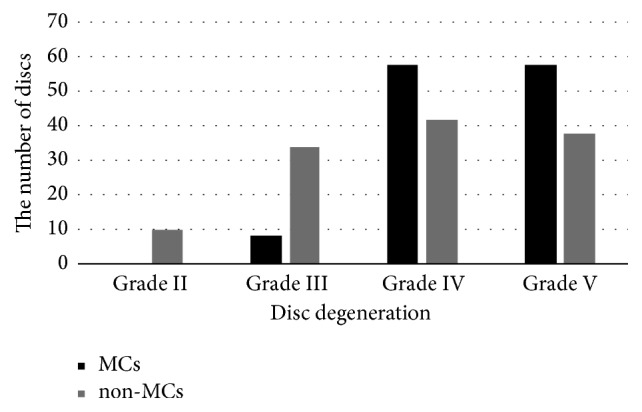
The number of discs with different degrees of degeneration. Of all the discs adjacent to type II MCs, more than 90% had grade IV and V degeneration. No healthy discs were found accompanied by type II MCs. There were less degenerative discs near non-MCs areas.

Adjacent to type II MCs, there were 8 (6.4%) discs with grade III degeneration, 58 (46.8%) with grade IV, and 58 (46.8%) with grade V. However, there were less degenerative discs near non-MCs areas (Figure 3). CT values of MC regions and the ΔCT value adjacent to discs with different degrees of degeneration are presented in Tables 4 and 5. There was no significant difference in the CT value of the bone marrow area (277.87±108.77 HU versus 255.62±124.25 HU versus 265.77±123.08 HU, P=0.5597) or endplate (461.80±93.05 HU versus 480.59±123.04 HU versus 472.62±133.38 HU, P=0.7400) between the various degrees of disc degeneration. Nevertheless, the ΔCT value of grade V disc degeneration was larger than that of grade IV at the endplate (97.14±116.63 HU versus 41.96±117.56 HU, P=0.0150), while no difference was found between them at the bone marrow area (114.23±100.62 HU versus 90.16±103.93 HU, P=0.3746).

## 4. Discussion

### 4.1. The Characteristics of Distribution

As it is known to all, type II MCs are the most common among these three types and present in pairs. The distribution of type II MCs in our study was in accordance with past reports. A study of 561 subjects revealed that about three-quarters (74.5%) of endplates with MCs were in the lower lumbar spine (adjacent to L4/5 and L5/S1 discs) and were most frequently adjacent to the L5/S1 disc [13]. In our results, the trend was in the same direction but was even more pronounced: 107 (86.3%) type II MCs were in the lower lumbar spine, and half of the total were adjacent to L5/S1. These data suggest that the lower lumbar spine is more prone to type II MCs than other areas. Also, our results indicated that MCs were almost always mirrored.

### 4.2. Sclerosis in Type II MCs

Type II MCs were long regarded as fat deposition since their initial description by Modic et al., based on six histological samples [5, 6]. That idea was widely accepted, and little research has been conducted to investigate the pathological entity of MCs. Interestingly, the results of our study are somewhat different from others.

In our study, type I, type III and mixed-type MCs were excluded so that only the pure type II MCs were included to avoid the interference from the signal intensity of other pathological processes. The CT values for water, fat, and bone are well known, and our findings show that the CT values of MCs (bone marrow, 261.80±122.93 HU; endplate, 475.65±126.45 HU) were significantly higher than those of non-MCs (bone marrow, 157.28±56.21 HU; endplate, 402.96±109.89 HU), which implies that some other kinds of pathological process might coexist with fat deposition in type II MCs. In addition, the consistency between non-MCs and the L1 vertebra suggests the control group was valid. Kuisma et al. found that endplate sclerosis existed in all types of MCs, especially in mixed MCs [7]. Xu et al. also found that 20.6% of 34 sclerotic endplates presented type II MCs [14]. However, the ratio of sclerotic type II MCs was relatively lower than other types. We speculate that this was because of their visual definition of sclerosis on CT images (Yes or No). Our quantitative measurements provide more accurate information, and it could be inferred that the unknown pathological process might be osteosclerosis. On the other hand, Feng et al. discovered that inflammatory reactions existed in type II MCs. They found that the great variety of signal intensities of MCs was not suppressed in the MR fat suppression sequence and that a specific subtype of MCs was associated with more severe disc degeneration [10]. In fact, MCs and disc degeneration are two parallel pathological processes and are strongly associated with each other, which means that the more severe the disc degeneration, the later the stage of the MC [15–17]. Although there are few reports in the literature in which reactive osteosclerosis appeared in high T2W signals, a study on osteoblastoma by Shaikh et al. showed that reactive sclerosis was accompanied by marrow edema [18]. Therefore, the signal not suppressed by the MR fat suppression sequence in the report of Feng et al. perhaps represented reactive osteosclerosis essentially, rather than inflammation, similar to our results. It is clear that trabecular sclerosis could occur in any type of inflammation in bone, and in fact sclerosis is the destiny of all bone inflammations. Thus, when reactive type I MCs are converted into stable type II MCs, osteosclerosis occurs.

### 4.3. Disc Degeneration and Type II MCs

As mentioned, the relationship between MCs and disc degeneration was noticed when MCs were first reported [4–6]. Since then, multiple kinds of assessment of disc degeneration have been introduced [12, 19, 20].

In the present study, we measured disc degeneration qualitatively based on the Pfirrmann classification. As a result, almost all of the discs adjacent to type II MCs were classified into grade IV and V degeneration, leaving only 8 (6.4%) grade III. These findings clearly reflect a tight correlation between MCs and disc degeneration, as was described in previous reports [15, 21]. Regarding the result that no significant difference was found in the CT value of the bone marrow area (P=0.5597) or endplate (P=0.7400) between the various degrees of disc degeneration, we believe it was the identical stage of MCs that made CT values increase to the same level. However, there was a significant difference between grade IV and V disc degeneration in the ΔCT value of the endplate (P=0.0150). We suppose it is possible that the different degrees of disc degeneration determine the extent of CT value changes compared with non-MCs. Often, lesions appear in the endplate at first, so the difference was strong there but not in the bone marrow area yet.

### 4.4. Limitations

There were some limitations to our study. First, MRI and CT are not able to replace pathological research completely. While MRI reflected vertebral bone marrow elements, CT showed the dense mineralized bone more. Each examination might lose some information, so further investigations on histological samples are needed. Second, the detection signal bias should not be neglected, because we used clinical patients as samples. If asymptomatic volunteers had been included, we would have had more discs with less degeneration and earlier-stage MCs and their CT values would be different. Third, we defined non-MCs as the area with larger CT values, from the lower endplate of the superior vertebra or the upper endplate of the inferior vertebra, if both the upper and lower endplate of one vertebra had MCs simultaneously. It might slightly affect the comparison between MCs and non-MCs due to the difference in CT values of different vertebras, but this condition was relatively rare. Results showed MCs had larger CT values than non-MCs did even though we chose the area with larger CT values as non-MCs. Another limitation was that non-MCs adjacent to MCs as a control group might have some small lesions that were undetectable and perturbed outcomes. However, we compared non-MCs with the healthy L1 vertebra to ensure they were credible. Despite those shortcomings, we believe our results are important because they reveal another pathological process of type II MCs.

## 5. Conclusions

The CT values of type II MC regions and the comparisons with non-MCs suggest that not only fat deposition but also osteosclerosis may exist in type II MCs. The more severe the degeneration of the adjacent disc, the greater the degree of osteosclerosis. In the future, more pathologic studies are needed to illuminate the pathological entities of type II MCs.

## Figures and Tables

**Table 1:** Demography of patients.

Demography of Patients	
Sample Size of Patients	66
Number of Type II MCs	124
Gender (Male/Female)	36/30
Age	59.94 (41-81) years
BMI	25.05 (19.05-33.06) kg/m2

**Table 2:** The location of type II MCs.

Endplates	Lower L2/Upper L3	Lower L3/Upper L4	Lower L4/Upper L5	Lower L5/Upper S1
Number	1/1	7/8	21/20	33/33

**Table 3:** CT values of MC regions, adjacent non-MC regions, and the L1 vertebra.

CT value (HU)	MCs	Non-MCs	L1 vertebra
Bone marrow area	261.80±122.93	157.28±56.21^*∗*^	148.61±42.51^*∗*^
Endplate	475.65±126.45	402.96±109.89^*∗*^	391.33±72.43^*∗*^

*∗*: P<0.0001 (compared with MCs). A vertebra was divided into the MC region and the non‐MC region. CT values of the bone marrow area and endplate at each region, including the L1 vertebra, were calculated separately.

**Table 4:** CT values of MC regions adjacent to discs with different degrees of degeneration.

CT value (HU)	Grade III	Grade IV	Grade V
Bone marrow area	277.87±108.77	255.62±124.25	265.77±123.08
Endplate	461.80±93.05	480.59±123.04	472.62±133.38

No significant difference was found in the CT value of the bone marrow area (P=0.5597) or endplate (P=0.7400) between the various degrees of disc degeneration.

**Table 5:** CT value changes (ΔCT value) of the MC regions adjacent to discs with different degrees of degeneration.

ΔCT value (HU)	Grade III	Grade IV	Grade V
Bone marrow area	138.25±75.08	90.16±103.93	114.23±100.62
Endplate	118.10±102.48	41.96±117.56	97.14±116.63^*∗*^

*∗*: P=0.0150 (IV compared with V, two-way ANOVA and LSD test). The ΔCT value of the endplate was significantly different between grade IV and V disc degeneration. No significant difference was found in the ΔCT value of bone marrow area between the various degrees of disc degeneration (P=0.3746).

## Data Availability

The data used to support the findings of this study are included within the article.
